# Supplementary material for: Direct allele introgression into pure chicken breeds using Sire Dam Surrogate (SDS) mating
Source: Nat Commun. 2021 Jan 28;12:659. doi: 10.1038/s41467-020-20812-x (PMC7844028; doi:10.1038/s41467-020-20812-x)
Supplement: Supplementary file 1 — Supplementary information [file 41467_2020_20812_MOESM1_ESM.pdf]

Supplementary Information

Direct allele introgression into pure chicken breeds using Sire Dam Surrogate (SDS) mating

Maeve Ballantyne, Mark Woodcock, Dadakhalandar Doddamani, Tuanjun Hu, Lorna Taylor, Rachel J. Hawken, Mike J. McGrew

Supplementary Table 1. Injection and hatching of surrogate host chicks carrying donor PGCs

Donor PGC genotypes	PGCs injected per embryo	No. of expts	Genotype of sire mated with wildtype hens to produce host eggs	No. of host eggs injected	No. of eggs viable at day 18 incubation[&]	No. of chicks hatched (% viable eggs[$])	Hatched host chicks with a sterile genotype*
iCaspase9♀<br>aviCaspase9♀	3000	1	ZZ^{DDX4-}	22	18	12 (67%)	2 (17%)
PMEL17 Line6♀<br>*PMEL17* Line6♂	6000	2	iCaspase9	26	NA	13	6 (46%)
PMEL17 Line6♀<br>*PMEL17* Line6♂	6000	2	aviCaspase9	27	18	10 (56%)	5 (50%)
KRT75 LSX♀<br>LSX♂	4000	1	iCaspase9	23	19	7 (37%)	4 (57%)
KRT75 LSX♀<br>LSX♂	4000	1	iCaspase9/<br>iCaspase9	19	15	8 (53%)	8 (100%)

[&]Viability; no. of eggs with viable embryos at day 18 of incubation detected by candling
[$]Hatchability; % chicks hatched from viable day 18 eggs.
NA; not available.
*For *DDX4* host eggs; 25% of the recipient host eggs fathered by the ZZ^{DDX4-} sire will be the sterile Z^{DDX4-} *W* genotype, 25% ZW, 25% ZZ and 25% ZZ^{DDX-}. For iCaspase9 host eggs; an iCaspase9 heterozygote male was initially bred to wildtype females should transmit the transgene to 50% of the recipient host eggs. A homozygous iCaspase9 male was subsequently generated which should transmit the iCaspase9 transgene to 100% of the recipient host eggs.

Supplementary Table 2. Germline transmission rates of *DDX4* surrogate hens carrying donor iCaspase9 targeted PGCs

Host genotype	No. of surrogates	No. of eggs laid per week*	No. of eggs incubated	Fertility[$] (% eggs incubated)	No. of chicks hatched[†] (% fertile eggs)	No. of transgenic chicks (% Transmission[ƻ])
$Z^{DDX4-}W$	2	3.33	85	80 (94%)	74 (93%)	8 (22%)
ZW	2	6.77	135	115 (85%)	93 (81%)	0 (0%)

*Lay rate; eggs in pen were counted over a 60 day period when hens were between 7-9 months of age and divided by the number of fertile hens present in pen. The maximum possible lay rate is 7.0 eggs per week.

[$]Fertility; no. of embryos detected at day 18 of incubation by candling.

[†]Hatchability; no. of chicks hatched from fertile day 18 eggs.

[ƻ]Transmission rate; the no. of iCaspase9 chicks per no. of hatched chicks equals one half the transmission rate due to meiotic reduction.

Supplementary Table 3. Lay rate of iCaspase9 heterozygote hens.

Hen genotype	No. of hens	No. of eggs laid per week*
iCaspase9	7	6.77
Wildtype	15	6.64

*Lay rate; eggs in pen were counted over a 60 day period when hens were between 7-9 months of age and divided by the number of fertile hens present in pen. The maximum possible lay rate is 7.0 eggs per week.

Supplementary Figures

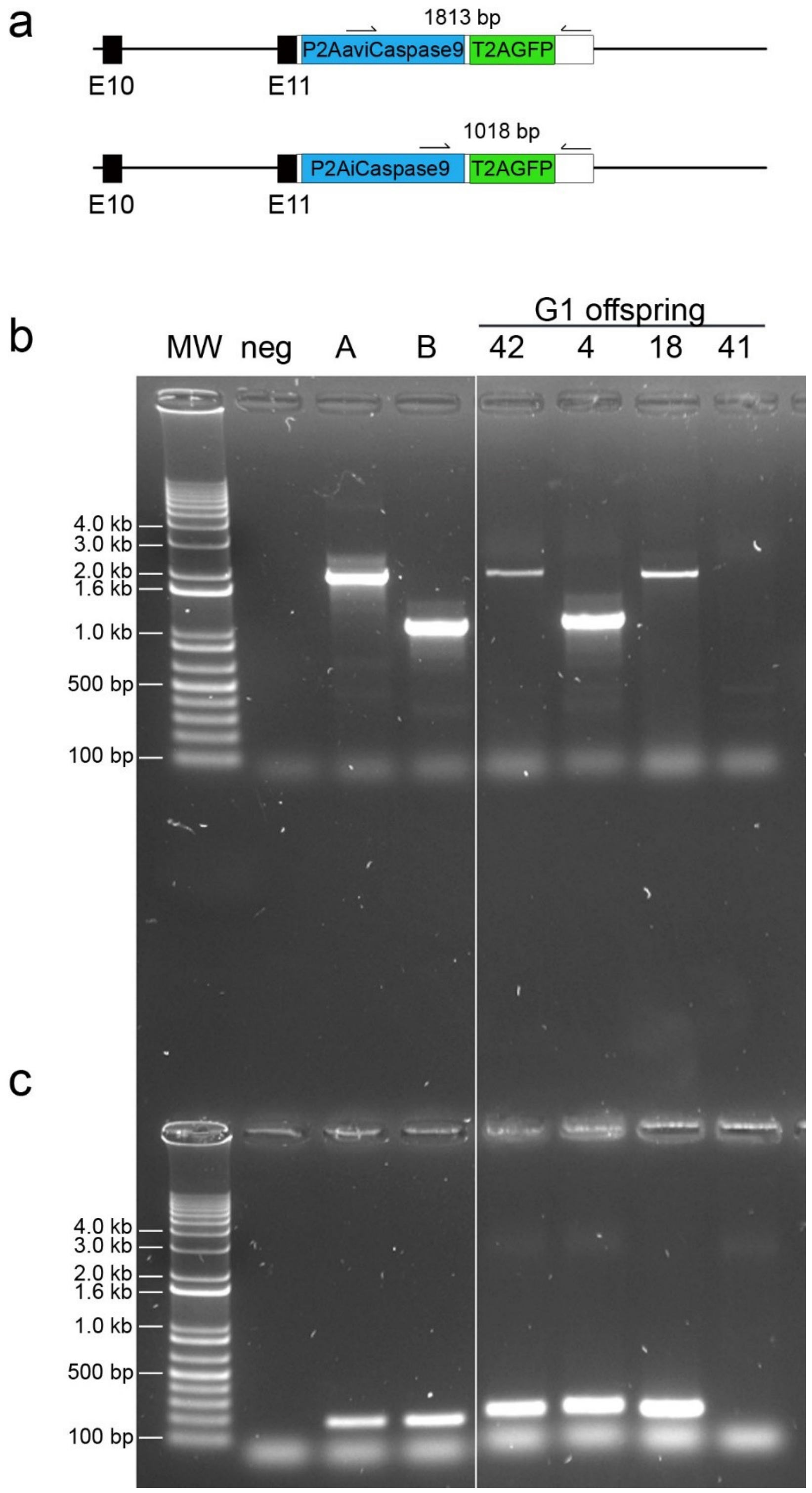

Supplementary Fig. 1: Generation of iCaspase9 and aviCaspase9 G_1 offspring.

a, Diagram of targeted *DAZL* locus and primer sites. **b,** PCR of G_1 offspring with Caspase9 specific primers; MW, molecular weight markers, A, targeted PGCs containing aviCaspase9 transgene, B, targeted PGCs containing iCaspase9 transgene. The aviCaspase9 transgene

produces a PCR product of 1813 bp. The iCaspase9 transgene produces a PCR product of 1013 bp. Positive offspring shown here are numbers 42, 18: aviCaspase9; 4: iCaspase9. **c,** PCR with primers specific for GFP, product size, 187 bp.

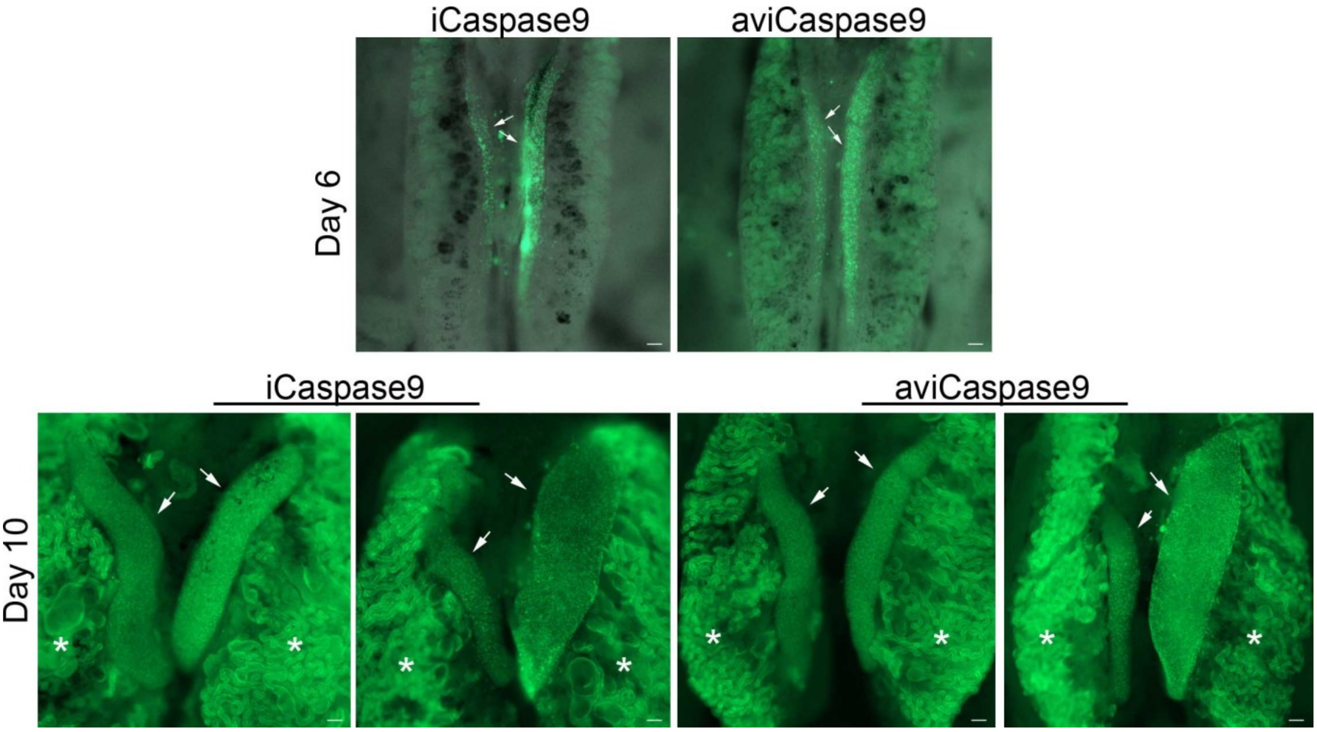

Supplementary Fig. 2: GFP expression in iCaspase9 and aviCaspase9 G_2 chicken embryos.

Representative gonads from day 6 and day 10 incubated G_2 embryos, PCR positive for the iCaspase9 or aviCaspase9 transgenes, were isolated and imaged for GFP fluorescence. Arrows indicate the embryonic gonads. *, autofluorescence in the underlying mesonephros. Scale bar, 100 μm.

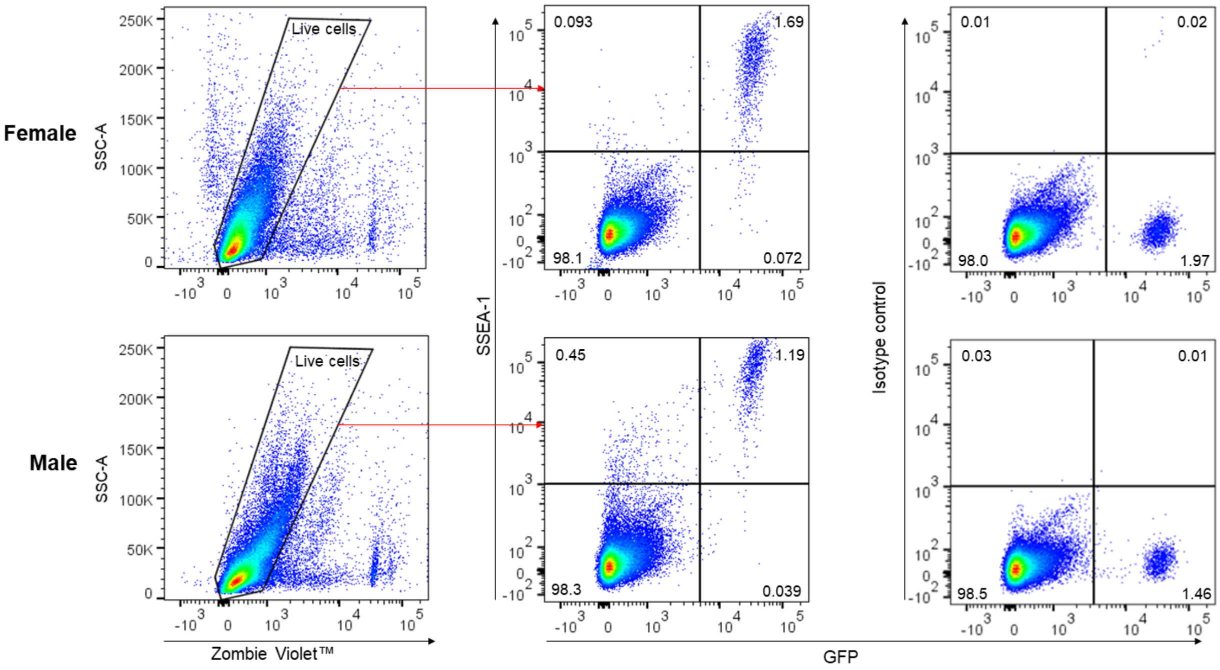

Supplementary Fig 3: Flow cytometric analysis of iCaspase9 embryonic gonadal PGCs. Gonads from one male and one female iCaspase embryos at day 10 of incubation were dissociated and stained with dead cell marker Zombie Violet and PGC marker, SSEA-1. The gated live cells were analysed for GFP and SSEA-1 expression; the results indicate that the majority (95.9% and 98.7%, for the ♀ and ♂ embryos respectively) of the GFP^{+} cell population also express SSEA-1 antigen, n = 4 male biological replicates, 4 female biological replicates . The gated live cells were analysed with an isotype control to determine the non-specific background signal. This is the same gating strategy and data that is presented in Fig. 1c.

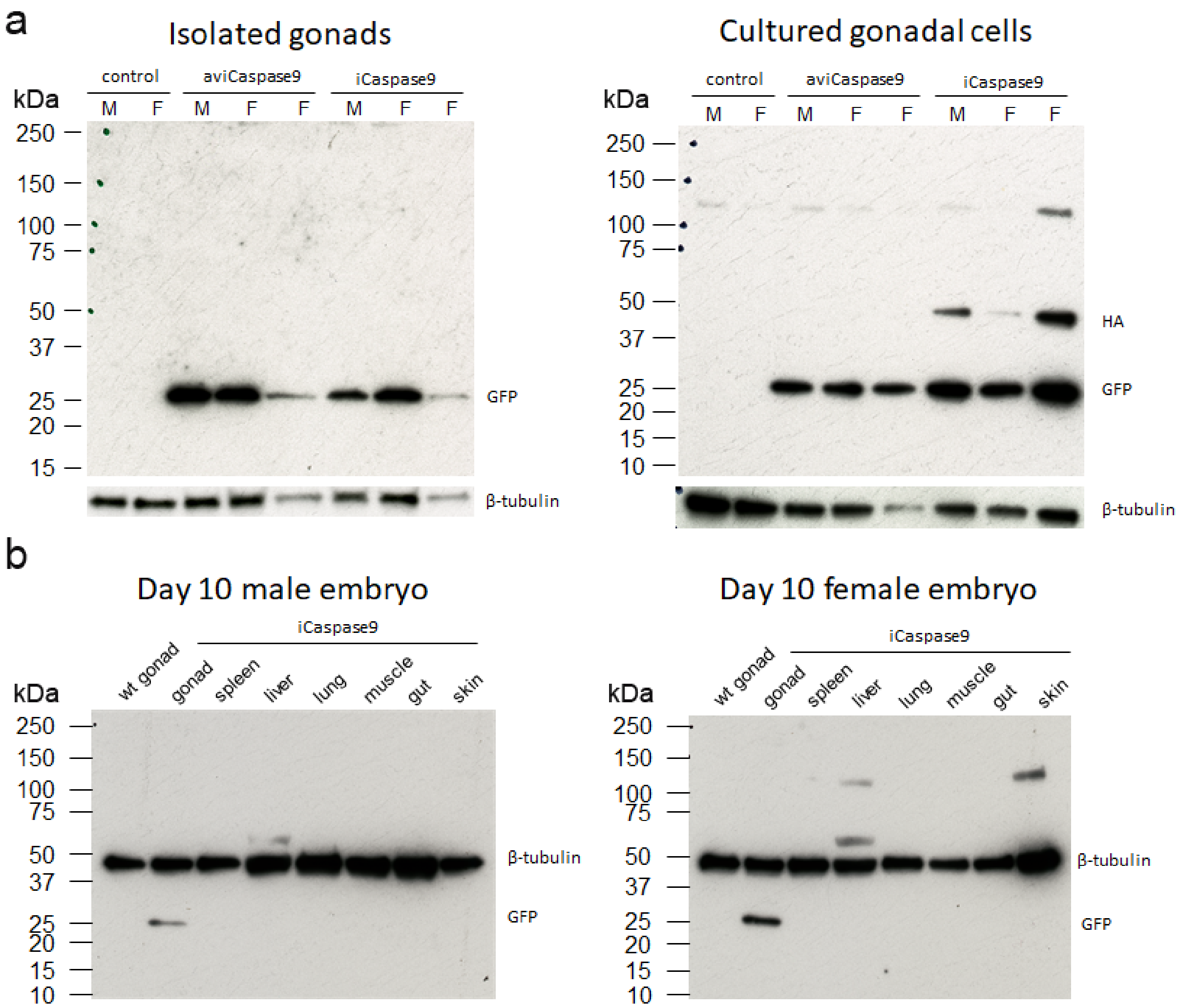

Supplementary Fig 4: Western blot analysis of iCaspase9 and aviCaspase9 embryonic gonads and tissues.

a, GFP$^+$ gonads from individual iCaspase9 or aviCaspase9 day 10 embryos were examined for GFP or HA-tag expression. Each gonadal sample was dissociated and one half immediately processed for Western blot analysis and the other half cultured for five days prior to lysing for Western blot analysis. Control = GFP$^-$ gonads. n = one blot each containing three transgenic biological replicates. **b**, Tissues from a single male and female iCaspase9 embryo were examined for GFP expression.

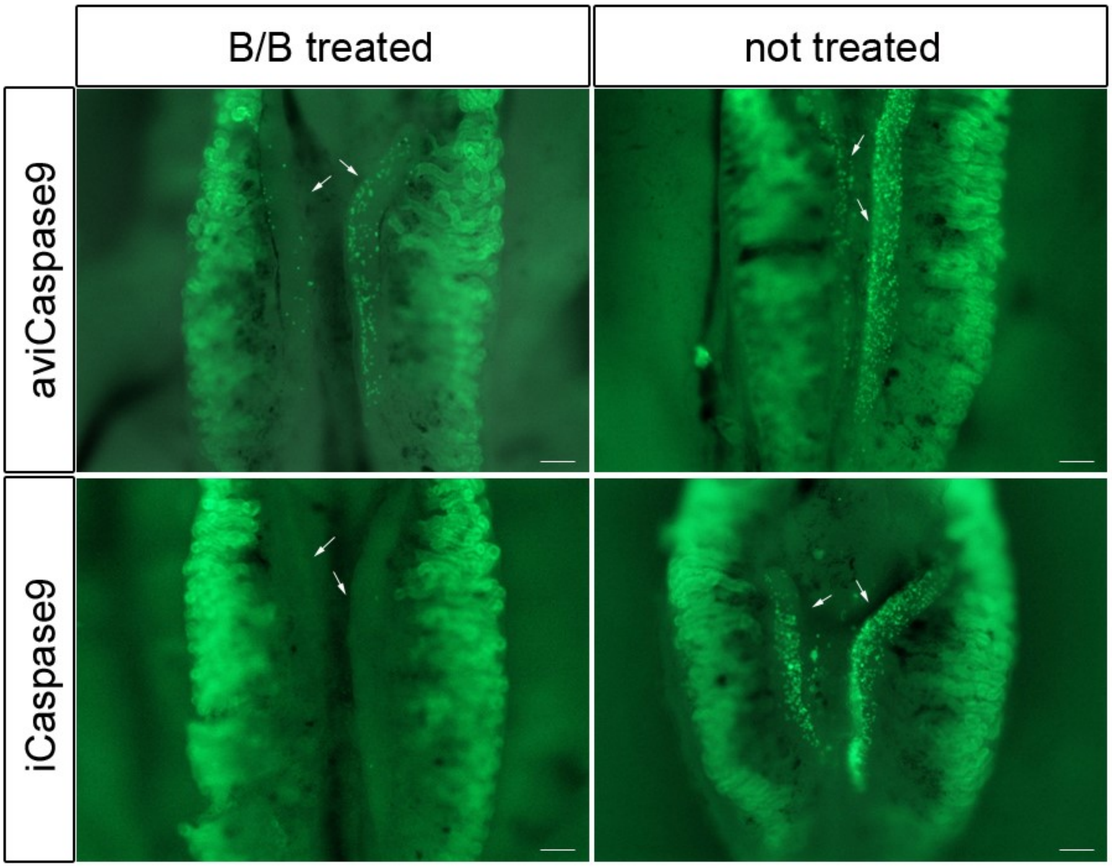

Supplementary Fig. 5: iCaspase9 embryos were more responsive to B/B compound than aviCaspase9 embryos.

Stage 16 HH (day 2.5) embryos were injected with 1μl of 0.1mM B/B compound into the dorsal aorta and incubated to day 6 of embryonic development. Arrows indicate the GFP^+ cells in the gonads. Fewer GFP^+ cells are present in the iCaspase9 embryo compared to the aviCaspase9 embryo post B/B treatment (n= 3, aviCaspase9; n = 5, iCaspase9). Scale bar, 100 μm.

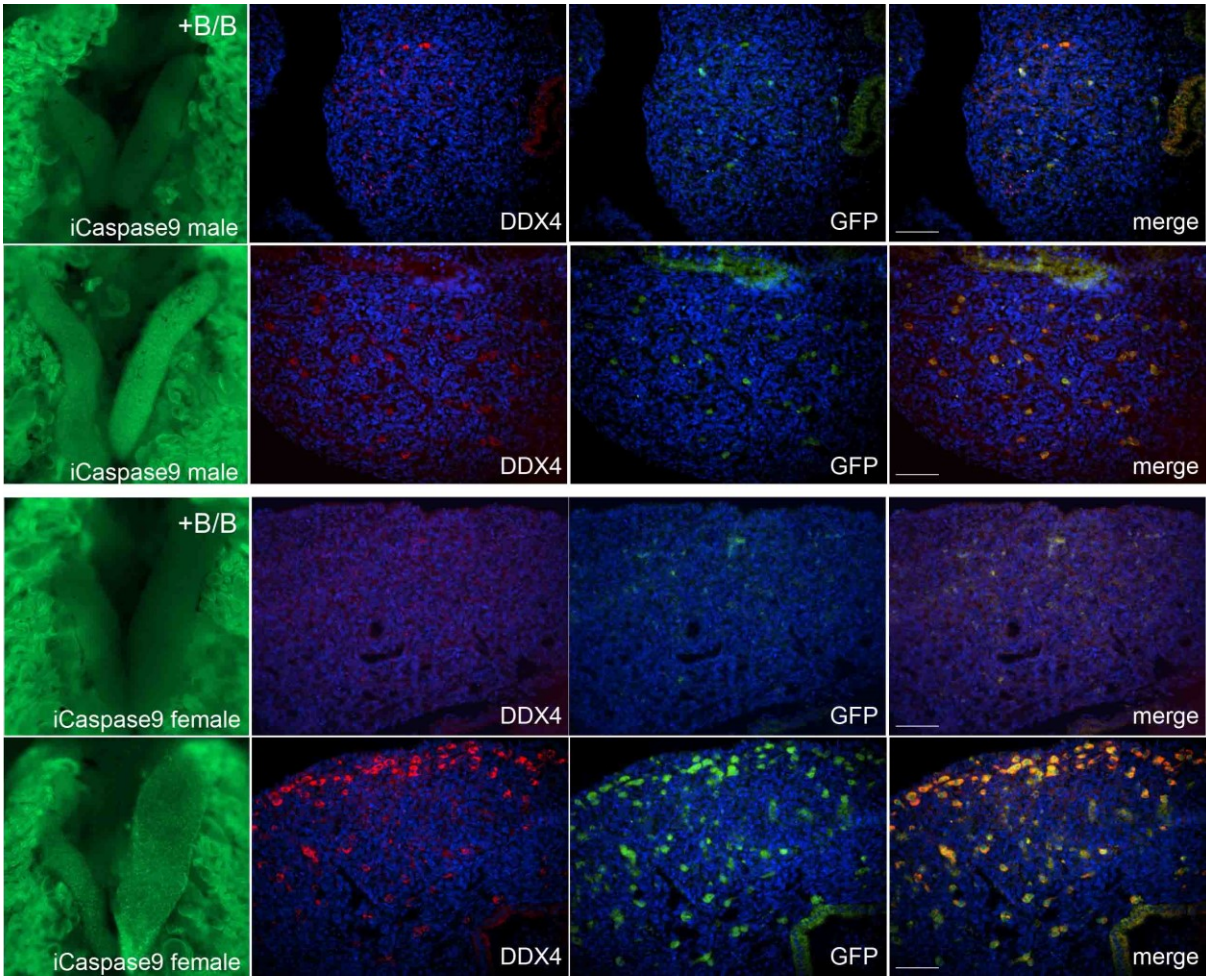

Supplementary Fig. 6: GFP expression is germ cell specific in day 10 iCaspase9 G_2 embryos and B/B treatment ablates endogenous PGCs.

Stage 16 HH (day 2.5) embryos were injected with 1 μl of 0.5 mM B/B compound into the dorsal aorta and incubated to day 10 of embryonic development. Cryosections were examined for endogenous GFP fluorescence (green) and DDX4 immunofluorescence (red). N = 3 for B/B treated embryos. Scale bar, 50 μm.

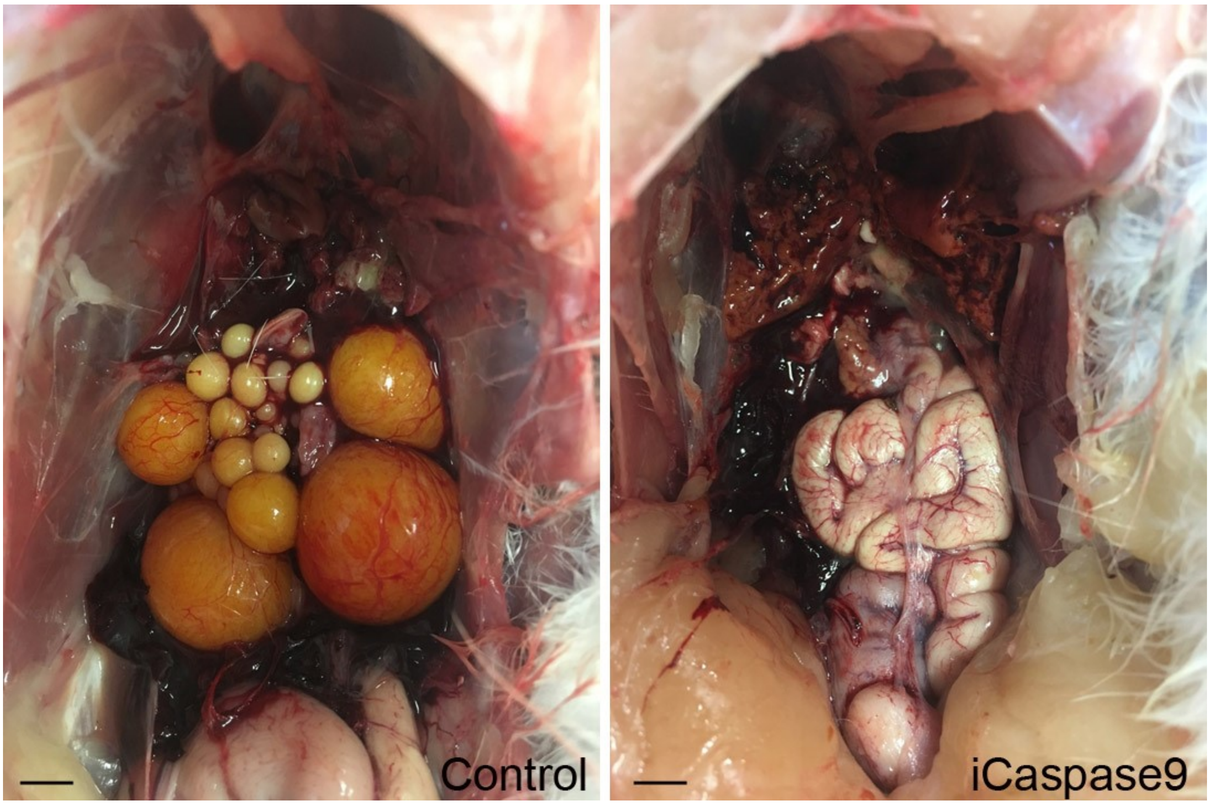

Supplementary Fig. 7: Oocyte formation in B/B treated iCaspase9 female embryos.

Wildtype control and iCaspase9 embryos were microinjected with 1 μl of 0.5 mM B/B compound into the dorsal aorta at stage 16 HH (day 2.5). Embryos were incubated, hatched and raised to 31 weeks of age. The control hen contains a normal follicular hierarchy. The iCaspase9 hen contains no follicles. Scale bar, 1 cm.

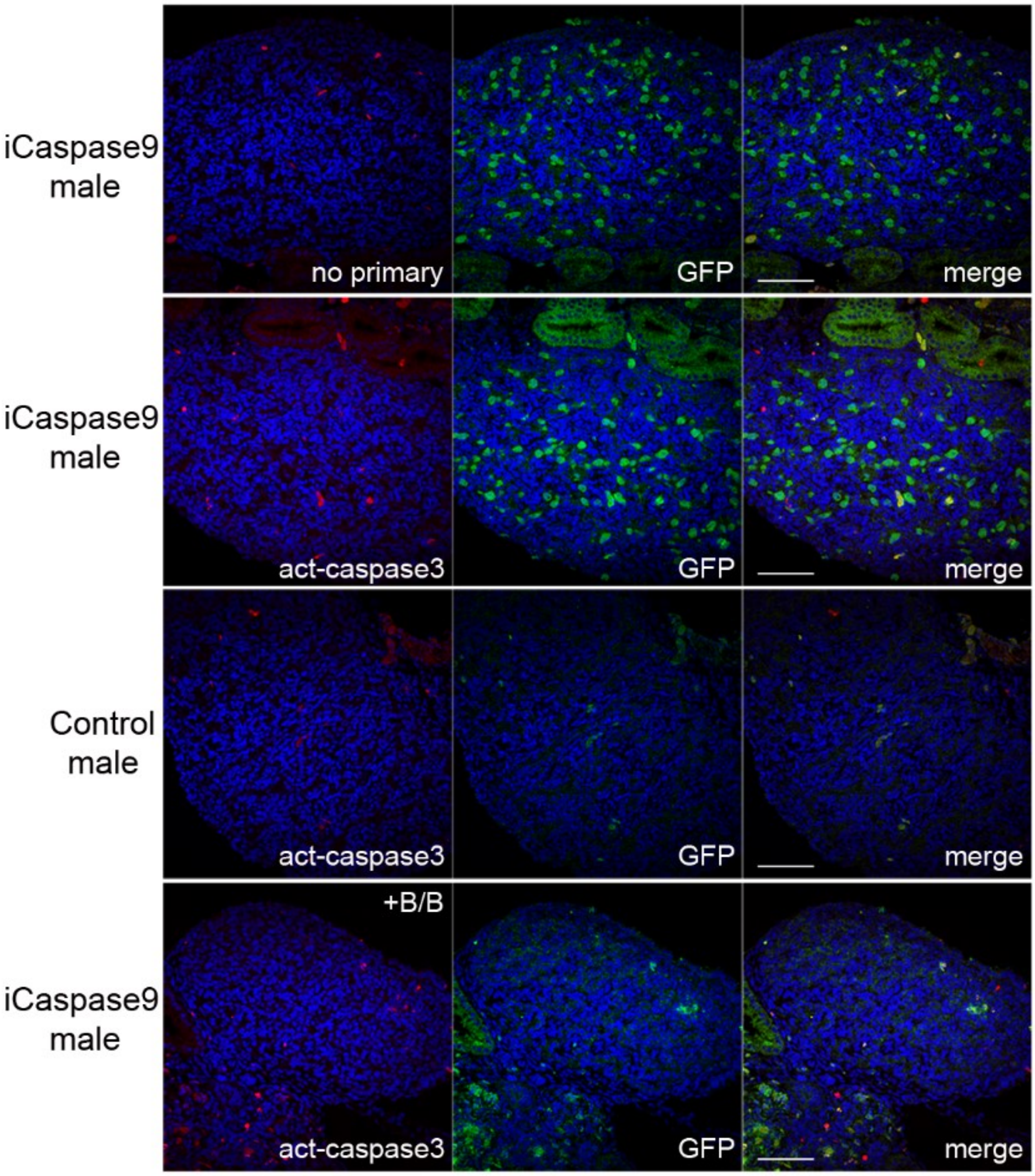

Supplementary Fig. 8: Activated caspase3 is not increased in day 10 iCaspase9 embryonic gonads.

Stage 16 HH (day 2.5) embryos were injected with 1 μl of 0.5mM B/B compound and incubated to day 10 of embryonic development. Cryosections were examined for endogenous GFP fluorescence (green) and activated caspase3 immunofluorescence (red). N = 3 male embryos, 1 control embryo. Size marker, 50 μm.

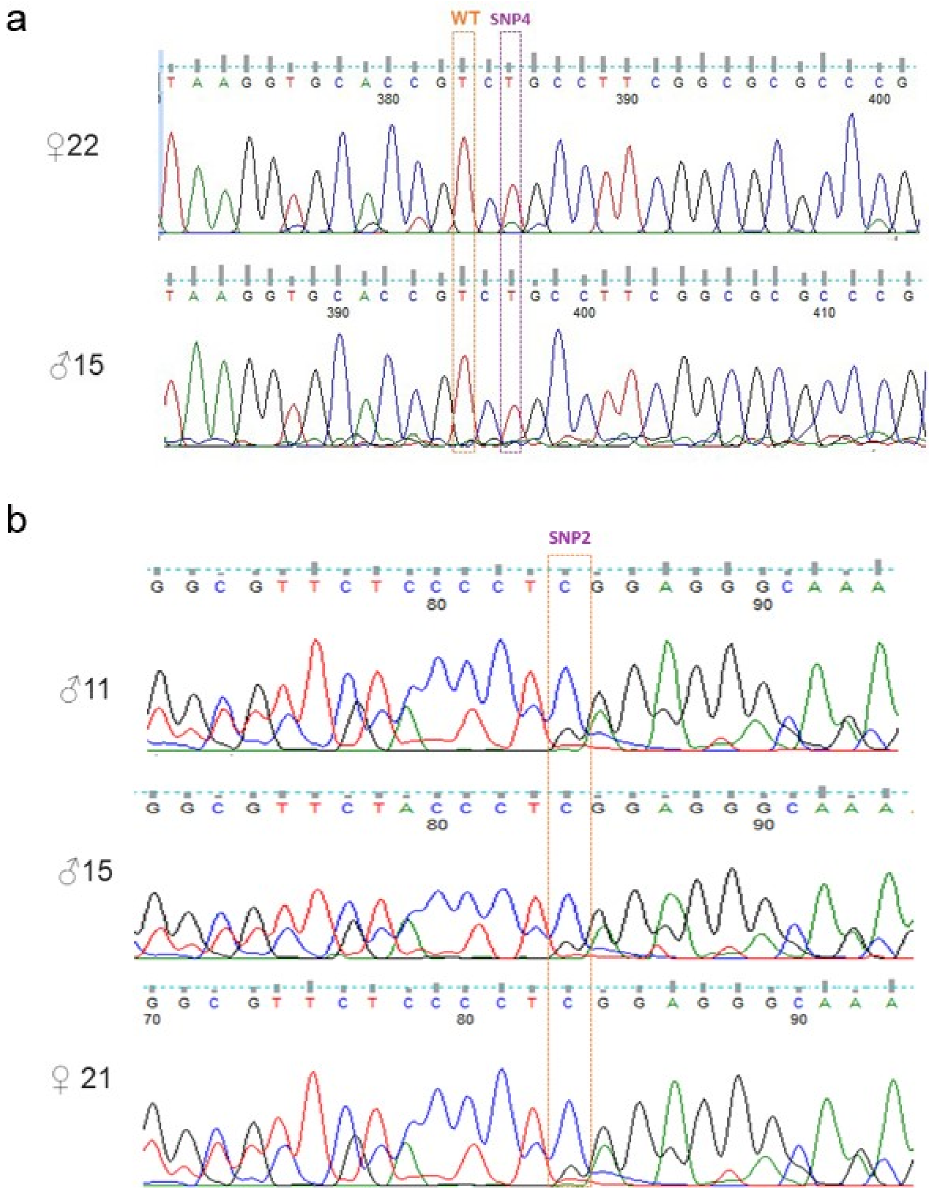

Supplementary Fig. 9: Sex-linked barring allele B2 is present in WL Line 6 chicken. Screening of individual WL Line 6 chicken for mutations in the Z chromosome *CDKN2A* gene **a,** WL Line 6 were homozygous for missense SNP4 R10C. Missense SNP3 associated with sex-linked barred allele B3 was not identified (boxed and labelled as WT). **b,** WL Line 6 were homozygous for non-coding SNP2.

a

OFF-TARGET NUMBER	SEQUENCE	CFD SCORE
PMEL17	GCTCACCGTGGGGCTGCTCT *GGG*	1.00
1	GCTCACC**AG**GGG**A**CT**A**CTCT *GGG*	0.571
2	GC**AT**A**T**C**A**TGGGGCTGCTCT *GGG*	0.531
3	**C**CTCAC**A**G**CA**GGGCTGCTCT *TGG*	0.462
4	**A**CTCAC**T**G**G**GGGGCTGCTCT *TGG*	0.453
5	GCT**G**A**A**C**A**TGG**A**GCTGCTCT *TGG*	0.433

b

***PMEL17* gRNA**

OFF-TARGET NUMBER

1 = intron:ENSGALT00000075913.1/ENSGALT00000055057.1 (Gallus_gallus-5.0, chr15:12,372,555-12,372,577)
2 = intergenic:FGFR3-ENSGALT00000073982.1/ENSGALT00000064614.1 (Gallus_gallus-5.0, chr4:84,180,725-84,180,747)
3 = intergenic:MTBP/MRPL13-MTBP (Gallus_gallus-5.0, chr2:137,045,597-137,045,619)
4 = intergenic:PI4KA-UBE2L3 (Gallus_gallus-5.0, chr15:178,914-178,936)
5 = intron:SCARA5 (Gallus_gallus-5.0, chr3:105,590,356-105,590,378)

c

OFF-TARGET 1 (intron:ENSGALT00000075913.1)

```
NCBI Gallus gallus (5.0)      TGCCAAGACTCCAGCACTACTAATGAAGCCATCTATATGGTCTGGAGA-GAGTCAAGAGTGAGGCTAGGAAGGGATGGATTGTGCTCTCTCAAATTCTTAGATC
WL control ♂                  ---------------------------------------------------TCAAGAGTGAGGCTAGGAAGGGATGGATTGTGCTCTCTCAAATTCTTAGATC
G1 DOW-H9                     ---------------------------------------------------TCAAGAGTGAGGCTAGGAAGGGATGGATTGTGCTCTCTCAAATTCTTAGATC
G1 DOW-H12                    ---------------------------------------------------TCAAGAGTGAGGCTAGGAAGGGATGGATTGTGCTCTCTCAAATTCTTAGATC
PMEL17 edited WL Clone.11 ♂   ---------------------------------------------------TCAAGAGTGAGGCTAGGAAGGGATGGATTGTGCTCTCTCAAATTCTTAGATC

NCBI Gallus gallus (5.0)      ATACACCATCATTTTACAAATGGCCCCACAATTCTTCAAAAAATGGCTCCAAATTGCACAGAACGTGTTCAGTTTTGGAGTTTCCAAGCTCCTAACCAACTATT
WL control ♂                  ATACACCATCATTTTACAAATGGCCCCACAATTCTTCAAAAAATGGCTCCAAATTGCACAGAACGTGTTCAGTTTTGGAGTTTCCAAGCTCCTAACCAACTATT
G1 DOW-H9                     ATACACCATCATTTTACAAATGGCCCCACAATTCTTCAAAAAATGGCTCCAAATTGCACAGAACGTGTTCAGTTTTGGAGTTTCCAAGCTCCTAACCAACTATT
G1 DOW-H12                    ATACACCATCATTTTACAAATGGCCCCACAATTCTTCAAAAAATGGCTCCAAATTGCACAGAACGTGTTCAGTTTTGGAGTTTCCAAGCTCCTAACCAACTATT
PMEL17 edited WL Clone.11 ♂   ATACACCATCATTTTACAAATGGCCCCACAATTCTTCAAAAAATGGCTCCAAATTGCACAGAACGTGTTCAGTTTTGGAGTTTCCAAGCTCCTAACCAACTATT

NCBI Gallus gallus (5.0)      TAGGCACAGTGACCTTTATCACATGTGGGGATCAGGGTTGATGCATGCAGTGTGCGCCCAGAGCTCACCAGGGGACTACTCTGGGACCAACTGACAGCATGGGA
WL control ♂                  TAGGCACAGTGACCTTTATCACATGTGGGGATCAGGGTTGATGCATGCAGTGTGCGCCCAGAGCTCACCAGGGGACTACTCTGGGACCAACTGACAGCATGGGA
G1 DOW-H9                     TAGGCACAGTGACCTTTATCACATGTGGGGATCAGGGTTGATGCATGCAGTGTGCGCCCAGAGCTCACCAGGGGACTACTCTGGGACCAACTGACAGCATGGGA
G1 DOW-H12                    TAGGCACAGTGACCTTTATCACATGTGGGGATCAGGGTTGATGCATGCAGTGTGCGCCCAGAGCTCACCAGGGGACTACTCTGGGACCAACTGACAGCATGGGA
PMEL17 edited WL Clone.11 ♂   TAGGCACAGTGACCTTTATCACATGTGGGGATCAGGGTTGATGCATGCAGTGTGCGCCCAGAGCTCACCAGGGGACTACTCTGGGACCAACTGACAGCATGGGA
```

OFF-TARGET 2 (intergenic:FGFR3)

```
NCBI Gallus gallus (5.0)      GAGATGAGCCCAGCACAATGCCCGTTTGCTAAAGAGAGATTTCCAAAGATGAAACAATCTTCCTGGGACAGTCTCACAGGGGTAAATGTGACGCAGCCAGCATG
WL control ♂                  ----------------------------------------------GATGAAACAATCTTCCTGGGACAGTCTCACAGGGGTAAATGTGACGCAGCCAGCATG
G1 DOW-H9                     ----------------------------------------------GATGAAACAATCTTCCTGGGACAGTCTCACAGGGGTAAATGTGACGCAGCCAGCATG
G1 DOW-H12                    ----------------------------------------------GATGAAACAATCTTCCTGGGACAGTCTCACAGGGGTAAATGTGACGCAGCCAGCATG
PMEL17 edited WL Clone.11 ♂   ----------------------------------------------GATGAAACAATCTTCCTGGGACAGTCTCACAGGGGTAAATGTGACGCAGCCAGCATG

NCBI Gallus gallus (5.0)      GGTAAATGTGACGCAGCCAGCATGGATATGGAGCTTCATCTGGGAGGAGAAGTGCAGCGTGCTCAGCCCAGAGCAGCCCCATGATATGCATCTTGCACACACAT
WL control ♂                  GGTAAATGTGACGCAGCCAGCATGGATATGGAGCTTCATCTGGGAGGAGAAGTGCAGCGTGCTCAGCCCAGAGCAGCCCCATGGTATGCATCTTGCACACACAT
G1 DOW-H9                     GGTAAATGTGACGCAGCCAGCATGGATATGGAGCTTCATCTGGGAGGAGAAGTGCAGCGTGCTCAGCCCAGAGCAGCCCCATGGTATGCATCTTGCACACACAT
G1 DOW-H12                    GGTAAATGTGACGCAGCCAGCATGGATATGGAGCTTCATCTGGGAGGAGAAGTGCAGCGTGCTCAGCCCAGAGCAGCCCCATGGTATGCATCTTGCACACACAT
PMEL17 edited WL Clone.11 ♂   GGTAAATGTGACGCAGCCAGCATGGATATGGAGCTTCATCTGGGAGGAGAAGTGCAGCGTGCTCAGCCCAGAGCAGCCCCATGGTATGCATCTTGCACACACAT
```

OFF-TARGET 3 (intergenic:MTBP/MRPL13-MTBP)

```
NCBI Gallus gallus (5.0)      TTGCCTGGGGTTGTTGTGATCAAAATGCAGGACCCGACACTTGGCCCTATTGAAACTCATA-CAAGTTACCTTGGCCCACTGATCCAGCCTATCCAGGTCCCTC
WL control ♂                  ---------------------------------------------------------------AAGTTACCTTGGCCCACTGATCCAGCCTATCCAGGTCCCTC
G1 DOW-H9                     ---------------------------------------------------------------AAGTTACCTTGGCCCACTGATCCAGCCTATCCAGGTCCCTC
G1 DOW-H12                    ---------------------------------------------------------------AAGTTACCTTGGCCCACTGATCCAGCCTATCCAGGTCCCTC
PMEL17 edited WL Clone.11 ♂   ---------------------------------------------------------------AAGTTACCTTGGCCCACTGATCCAGCCTATCCAGGTCCCTC

NCBI Gallus gallus (5.0)      TGTAGCACCTTTCTCCCCTCAGACAGATCAACACTCTATCCCAACTTGATGTTGTCTACAAATTTACTGAGGGTGCACTTAATCCCTTCAGCAAGATCATTAAT
WL control ♂                  TGTAGCACCTTTCTCCCCTCAGACAGATCAACACTCTATCCCAACTTGATGTTGTCTACAAATTTACTGAGGGTGCACTCAATCCCTTCATCAAGATCATTAAT
G1 DOW-H9                     TGTAGCACCTTTCTCCCCTCAGACAGATCAACACTCTATCCCAACTTGATGTTGTCTACAAATTTACTGAGGGTGCACTCAATCCCTTCATCAAGATCATTAAT
G1 DOW-H12                    TGTAGCACCTTTCTCCCCTCAGACAGATCAACACTCTATCCCAACTTGATGTTGTCTACAAATTTACTGAGGGTGCACTCAATCCCTTCATCAAGATCATTAAT
PMEL17 edited WL Clone.11 ♂   TGTAGCACCTTTCTCCCCTCAGACAGATCAACACTCTATCCCAACTTGATGTTGTCTACAAATTTACTGAGGGTGCACTCAATCCCTTCATCAAGATCATTAAT

NCBI Gallus gallus (5.0)      AAAGACACCGAACATAAGCCAGCAGCGTGCCCTCGCAGTAAAGAAAGCCAAAGAGTCCCATGGAGGATAACCAAGAGCAGCCCTGCTGTGAGGCTGAGAGCTGA
WL control ♂                  AAAGACACCGAACATAAGCCAGCAGCACGCCCTCGCAGTAAAGAAAGCCAAAGAGTCCCATGGAGGATAACCAAGAGCAGCCCTGCTGTGAGGCTGAGAGCTGA
G1 DOW-H9                     AAAGACACCGAACATAAGCCAGCAGCACGCCCTCGCAGTAAAGAAAGCCAAAGAGTCCCATGGAGGATAACCAAGAGCAGCCCTGCTGTGAGGCTGAGAGCTGA
G1 DOW-H12                    AAAGACACCGAACATAAGCCAGCAGCACGCCCTCGCAGTAAAGAAAGCCAAAGAGTCCCATGGAGGATAACCAAGAGCAGCCCTGCTGTGAGGCTGAGAGCTGA
PMEL17 edited WL Clone.11 ♂   AAAGACACCGAACATAAGCCAGCAGCACGCCCTCGCAGTAAAGAAAGCCAAAGAGTCCCATGGAGGATAACCAAGAGCAGCCCTGCTGTGAGGCTGAGAGCTGA
```

OFF-TARGET 4 (intergenic:PI4KA-UBE2L3)

```
NCBI Gallus gallus (5.0)      GAGCTGGAGGGGTACCATACTGATATAACATGCTGTAGGGGAACAAACAAAGAATCAGTAGTTGCACATCTTTCACTTAGTGTATCAGTTTGCTAAGTCTTTTA
WL control ♂                  -----------------------------------------------AAGAATCAGTAGTTGCACATCTTTCACTTAGTGTATCAGTTTGCTAAGTCTTTTA
G1 DOW-H9                     -----------------------------------------------AAGAATCAGTAGTTGCACATCTTTCACTTAGTGTATCAGTTTGCTAAGTCTTTTA
G1 DOW-H12                    -----------------------------------------------AAGAATCAGTAGTTGCACATCTTTCACTTAGTGTATCAGTTTGCTAAGTCTTTTA
PMEL17 edited WL Clone.11 ♂   -----------------------------------------------AAGAATCAGTAGTTGCACATCTTTCACTTAGTGTATCAGTTTGCTAAGTCTTTTA

NCBI Gallus gallus (5.0)      TGGCCCTGCACCTAGCAACAGGATGAGATGGACTTGCTACAGGCAAACAGCAAGCCTGCCAAGAGCAGCCCCCCAGTGAGTTCAGCAATGTGCTACTGTGGAGC
WL control ♂                  TGGCCCTGCACCTAGCAACAGGATGAGATGGACTTGCTACAGGCAAACAGCAAGCCTGCCAAGAGCAGCCCCCCAGTGAGTTCAGCAATGTGCTACTGTGGAGC
G1 DOW-H9                     TGGCCCTGCACCTAGCAACAGGATGAGATGGACTTGCTACAGGCAAACAGCAAGCCTGCCAAGAGCAGCCCCCCAGTGAGTTCAGCAATGTGCTACTGTGGAGC
G1 DOW-H12                    TGGCCCTGCACCTAGCAACAGGATGAGATGGACTTGCTACAGGCAAACAGCAAGCCTGCCAAGAGCAGCCCCCCAGTGAGTTCAGCAATGTGCTACTGTGGAGC
PMEL17 edited WL Clone.11 ♂   TGGCCCTGCACCTAGCAACAGGATGAGATGGACTTGCTACAGGCAAACAGCAAGCCTGCCAAGAGCAGCCCCCCAGTGAGTTCAGCAATGTGCTACTGTGGAGC
```

OFF-TARGET 5 (intron:SCARA5)

```
NCBI Gallus gallus (5.0)      GCAGGGGACATCCATGAACACTCACCCATGTCACTGCCTCGGTCCCCCTTCTCTCCCTTTGGTCCTCGATCACCTGG-TAAGAGAAAGAGTACGGGATATCAGG
WL control ♂                  -------------------------------------------TCCCCTTCTCTCCCTTTGGTCCTCGATCACCTGGGTAAGAGAAAGAGTACGGGATATCAGG
G1 DOW-H9                     -------------------------------------------TCCCCTTCTCTCCCTTTGGTCCTCGATCACCTGG-TAAGAGAAAGAGTACGGGATATCAGG
G1 DOW-H12                    -------------------------------------------TCCCCTTCTCTCCCTTTGGTCCTCGATCACCTGG-TAAGAGAAAGAGTACGGGATATCAGG
PMEL17 edited WL Clone.11 ♂   -------------------------------------------TCCCCTTCTCTCCCTTTGGTCCTCGATCACCTGG-TAAGAGAAAGAGTACGGGATATCAGG

NCBI Gallus gallus (5.0)      AGAGAAAGAGTACGGGATATCAGGCTTGCTGAAAGGTGATATATCAGCCTCTAGCAAGGCAGTGGCTGCTGAACATGGAGCTGCTCTTGGGAAAAGAGCGGTTT
WL control ♂                  AGAGAAAGAGTACGGGATATCAGGCTTGCTGAAAGGAGATATATCAGCCTCTAGCAAGGCAGTGGCTGCTGAACATGGAGCTGCTCTTGGGAAAAGAGCGGTTT
G1 DOW-H9                     AGAGAAAGAGTACGGGATATCAGGCTTGCTGAAAGGAGATATATCAGCCTCTAGCAAGGCAGTGGCTGCTGAACATGGAGCTGCTCTTGGGAAAAGAGCGGTTT
G1 DOW-H12                    AGAGAAAGAGTACGGGATATCAGGCTTGCTGAAAGGAGATATATCAGCCTCTAGCAAGGCAGTGGCTGCTGAACATGGAGCTGCTCTTGGGAAAAGAGCGGTTT
PMEL17 edited WL Clone.11 ♂   AGAGAAAGAGTACGGGATATCAGGCTTGCTGAAAGGAGATATATCAGCCTCTAGCAAGGCAGTGGCTGCTGAACATGGAGCTGCTCTTGGGAAAAGAGCGGTTT
```

Supplementary Fig. 10: Analysis of potential off-target mutations of *PMEL17* gRNA.

a, List of five potential off-target sites with the highest CFD scores. The protospacer adjacent motifs (PAM) are in italics and mismatches between the gRNA and off-target sites are highlighted in bold. **b**, Genetic loci of potential gRNA off-target sites. **c**, Alignments of the potential off-target site sequences of a WL Line 6 control chicken, a *PMEL17*-edited WL Line 6 PGC clone, two *PMEL17*-edited G_1 offspring and sequence from the NCBI *Gallus gallus* (5.0) genome. The sequencing primer is in blue and the gDNA complementary sequence in red (with the mismatches from the PMEL17 gRNA sequence in bold and underlined). Any sequence changes from the NCBI reference genome sequence are shown in green.

a

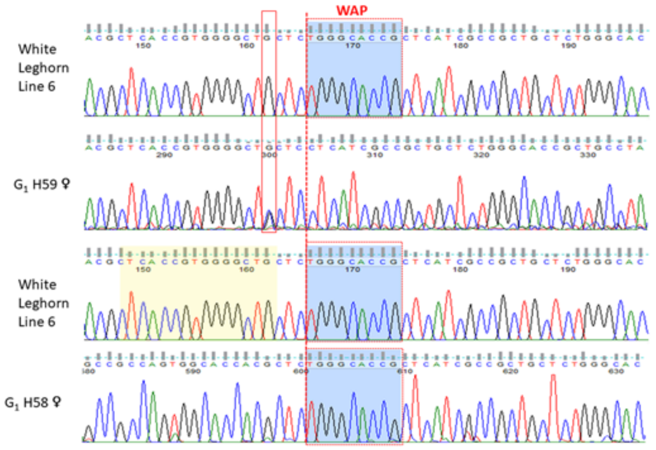

b

Genotype	number	phenotype
PMEL17 $^{-WAP/-WAP}$	9	6 barred
		3 speckled
PMEL17 $^{-WAP/Del+WAP}$	8	7 barred
		1 speckled
PMEL17 $^{Del+WAP/Del+WAP}$	3	2 barred
		1 speckled

c

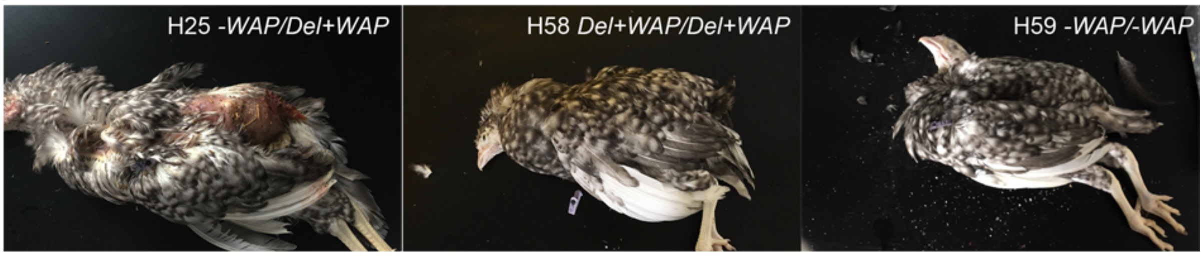

Supplementary Fig. 11: Sequence and feather phenotypes of *PMEL17* edited G_1 birds.

a, Sequence of G_1 offspring demonstrating the transmission of the edited *PMEL17* alleles. Bird H59 is homozygous for wildtype allele lacking the WAP insert, *PMEL17* $^{-WAP/-WAP}$. The red box indicates the tracking nucleotide (G->C) introduced into female WL PGCs. Bird H58 is homozygous for the novel 15 bp deletion (highlighted in yellow).

b, Number of female offspring for all feather phenotypes. Female birds contained barred or speckled feathers. The novel 15 bp deletion was underrepresented in the hatched offspring however one of the surrogate males carried *PMEL17* $^{-WAP/-WAP}$ edited PGCs and thus, would not generate *PMEL17* $^{Del+WAP}$ offspring.

c, Barred feathers in G_1 offspring. Similar feather colouring was present in all three genotypes.

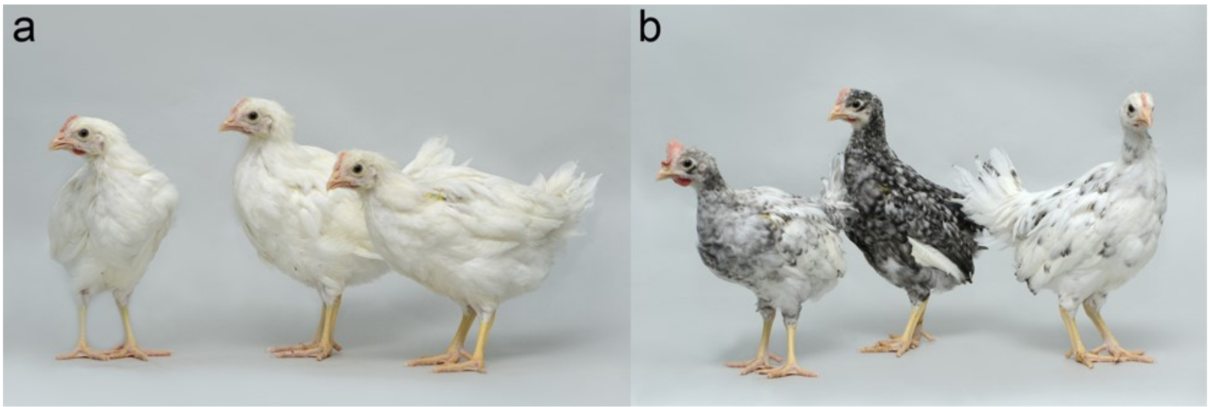

Supplementary Fig. 12: G_2 offspring from G1 *PMEL17* edited birds.
a, G_2 offspring from G_1H24♂ (*PMEL17*$^{-WAP/Del+WAP}$) mated to WL Line 6 control females
b, G_2 offspring from G_1H24♂ mated to G_1H9 (*PMEL17*$^{-WAP/-WAP}$) and G_1H12♀(*PMEL17*$^{-WAP/Del+WAP}$). All photographs of live birds at The Roslin Institute.

a

OFF-TARGET NUMBER	SEQUENCE	CFD SCORE
KRT75	GGCTTCAGCCCGGCTGCGGT *TGG*	1.000
1	**T**GC**C**TCAGCCCGGCTGCGGT *TGG*	0.647
2	**A**GCT**G**CAGCCCGGCTGCGGT *TGG*	0.576
3	G**A**CTTCA**A**CCC**A**GCTGCG**T**T *AGG*	0.526
4	GGCTTCA**A**CC**A**GGCTGC**T**GT *GGG*	0.500
5	**T**GCT**G**CAG**AA**CGGCTGCGGT *TGG*	0.475

b

***KRT75* gRNA**
OFF-TARGET NUMBER
1 = exon:KRT5 (Gallus_gallus-5.0, chr33:1,317,408-1,317,430)
2 = intergenic:LHX2-NEK6 (Gallus_gallus-5.0, chr17:9,768,189-9,768,211)
3 = intron:CUX1 (Gallus_gallus-5.0, chr19:3,923,423-3,923,445, 23)
4 = intergenic:PPP1R3A-GPR85 (Gallus_gallus-5.0, chr1:26,604,012-26,604,034 23)
5 = intron:TCF7 (Gallus_gallus-5.0, chr13:16,031,678-16,031,700, 23)

c

OFF-TARGET 1 (exon:KRT5)

```
NCBI Gallus gallus (5.0)        TCTCTTGTGCAGTGCTGAGATCCTTCTTTTGTGAGTCCCACCTCCCTTCAGCTAGCATGATGCAGCATCTGCGGTCCCAGTTACCCACCTTGTTCTGGTACCAAGCC
LSX WT male                     ----------------------------------------------TTCAGCTAGCATGATGCAGCATCTGCGGTCCCAGTTACCCACCTTGTTCTGGTACCAAGCC
FRZ edited LSX Clone.22 female  ----------------------------------------------TTCAGCTAGCATGATGCAGCATCTGCGGTCCCAGTTACCCACCTTGTTCTGGTACCAAGCC

NCBI Gallus gallus (5.0)        TCTGCCTCAGCCCGGCTGCGGTTGGCGATGTCCTCATACTGTGCCTTCACCTCTGCGATGATGCTGTTGAGGTCCAGGCTGCGGTTGTTGTCCATGGACAGCACCAC
LSX WT male                     TCTGCCTCAGCCCGGCTGCGGTTGGCGATGTCCTCATACTGTGCCTTCACCTCTGCGATGATGCTGTTGAGGTCAAGGCTGCGGTTGTTGTCCATGGACAGCACCAC
FRZ edited LSX Clone.22 female  TCTGCCTCAGCCCGGCTGCGGTTGGCGATGTCCTCATACTGTGCCTTCACCTCTGCGATGATGCTGTTGAGGTCAAGGCTGCGGTTGTTGTCCATGGACAGCACCAC
```

OFF-TARGET 2 (intergenic:LHX2-NEK6)

```
NCBI Gallus gallus (5.0)        TGCACTCTCTGCCCGATTAGCTTCTTGAAATTTGGTTTCAGTAAATGGGATGAGCAAGGCAGAAACTTCCAAAGAGATTTTCCTCACTGGCATTTGGAGGACGAGCC
LSX WT male                     ---------------------------------------------TGGGATGAGCAAGGCAGAAACTTCCAAAGAGATTTTCCTCACTGGCATTTGGAGGACGAGCC
FRZ edited LSX Clone.22 female  ---------------------------------------------TGGGATGAGCAAGGCAGAAACTTCCAAAGAGATTTTCCTCACTGGCATTTGGAGGACGAGCC

NCBI Gallus gallus (5.0)        ACAGCACAGAGCTCCTGGAGCTGCAGCCCGGCTGCGGTTGGTTCACCCTTCACTCAGGCCTAACGCAGGAAAAGCTCCGACATAAATTGCAGCAGAGAAACACCAGC
LSX WT male                     ACAGCACAGAGCTCCTGGAGCTGCAGCCCGGCTGCGGTTGGTTCACCCTTCACTCAGGCCTAAAGCAGGAAAAGCTCCGACATAAATTGCAGCAGAGAAACACCAGC
FRZ edited LSX Clone.22 female  ACAGCACAGAGCTCCTGGAGCTGCAGCCCGGCTGCGGTTGGTTCACCCTTCACTCAGGCCTAAAGCAGGAAAAGCTCCGACATAAATTGCAGCAGAGAAACACCAGC
```

OFF-TARGET 3 (intron:CUX1)

```
NCBI Gallus gallus (5.0)        CAGGGAGGGTTTTCCGTACCCTGCTGGAGAAGTGGACCAGACAGAGGTGGGCAGGAGCCCGTGCTGGTTCAGAGTCTGTGCTGAGGGCCATTAATGCACTTTATTT
LSX WT male                     -------------------------------------------GAGGTGGGCAGGAGCCCGTGCTGGTTCAGAGTCTGTGCTGAGGGCCATTAATGCACTTTATTT
FRZ edited LSX Clone.22 female  -------------------------------------------GAGGTGGGCAGGAGCCCGTGCTGGTTCAGAGTCTGTGCTGAGGGCCATTAATGCACTTTATTT

NCBI Gallus gallus (5.0)        GTTGTTCTTACCTTGAAGAATTCTGCCCTAACGCAGCTGGGTTGAAGTCCCAGATGAGGGGAAGATGGGACAGAAGGTTCAGGGGGTTCCGGCGGTCCGTACCTGCA
LSX WT male                     GTTGTTCTTACCTTGAAGAATTCTGCCCTAACGCAGCTGGGTTGAAGTCCCAGATGAGGGGAAGATGGGACAGAAGGTTCAGGGGGTTCCGGCGGTCCGTACCTGCA
FRZ edited LSX Clone.22 female  GTTGTTCTTACCTTGAAGAATTCTGCCCTAACGCAGCTGGGTTGAAGTCCCAGATGAGGGGAAGATGGGACAGAAGGTTCAGGGGGTTCCGGCGGTCCGTACCTGCA
```

OFF-TARGET 4 (intergenic:PPP1R3A-GPR85)

```
NCBI Gallus gallus (5.0)        GACGCCAGATAGTCAGAGCCTTAACAATCAGGATTGGAAAGCTAATTATATGCAAATAATTGG-AAAGGAAAAATTCAGTGTTGTGCAGGTGATTCTGTATATCAGAA
LSX WT male                     --------------------------ACCTNA--TGGAA--CTAATTATATGCAAATAATTGGGAAAGGAAAAATTCAGTGTTGTGCAGGTGATTCTGTATATCAGAA
FRZ edited LSX Clone.22 female  ---------------------------NCAGA--TGGAA--CTAATTATATGCAAATAATTGG-AAAGGAAAAATTCAGTGTTGTGCAGGTGATTCTGTATATCAGAA

NCBI Gallus gallus (5.0)        CAGAAGGCCAAGGGTTTGCACATGTCTCAGAATACAAATGCAGAATAGACACGGTTGCTTTGTTCAAGGGTCAGAAAAGGTGGTGAAGGAACTGGAATATGGGAGCAT
LSX WT male                     CAGAAGGCCAAGGGTTTGCACATGTCTCAGAATACAAATGCAGAATAGACACGGTTGCTTTGTTCAAGGGTCAGAAAAGGTAGTGAAGGAACTGGAATATGGGAGCAT
FRZ edited LSX Clone.22 female  CAGAAGGCCAAGGGTTTGCACATGTCTCAGAATACAAATGCAGAATAGACACGGTTGCTTTGTTCAAGGGTCAGAAAAGGTAGTGAAGGAACTGGAATATGGGAGCAT

NCBI Gallus gallus (5.0)        GGCAGGACTGCAGACATACTGGGCTTAAAGAGATGATTGACTAGGGAAAGCCTTACCTTTGGGGGAGGCACAGTTCTTGACAGAACAGATGGGCTTCAACCAGGCTGC
LSX WT male                     GGCAGGACTGCAGACATACTGGGCTTAAAGAGATGATTGACTAGGGAAAGCCTTACCTTTGGGGGAGGCACAGTTCTTGACAGAACAGATGGGCTTCAACCAGGCTGC
FRZ edited LSX Clone.22 female  GGCAGGACTGCAGACATACTGGGCTTAAAGAGATGATTGACTAGGGAAAGCCTTACCTTTGGGGGAGGCACAGTTCTTGACAGAACAGATGGGCTTCAACCAGGCTGC

NCBI Gallus gallus (5.0)        TGTGGGCTGTAACACAAACTGTAAGAAAGGCAGAAGGCTATGGCTCTACAAAGGTCTTTGTGGCTAATGCGTGG-
LSX WT male                     TGTGGGCTGTAACACAAACTGTAAGAAAGGCAGAAGGCTATGGCTCTACAAAGGTCTTTGTGGCTAATGCGTGG-
FRZ edited LSX Clone.22 female  TGTGGGCTGTAACACAAACTGTAAGAAAGGCAGAAGGCTATGGCTCTACAAAGGTCTTTGTGGCTAATGCGTGGG
```

OFF-TARGET 5 (intron:TCF7)

```
NCBI Gallus gallus (5.0)        GTCGTTCCTGCACTGCGATACTGCTCTGTAGGCATGCGGAGGCTGCGTGCACAGAGGTGTGGCGGCTGTGTTTTGTAGTGTAATTGATGCTCTGCGTAACCTCGGTTT
LSX WT male                     ------------------------------------------------------AGGTGTGGCGGCTGTGTTTTGTAGTGTAATTGATGCTCTGCGTAACCTCGGTTT
FRZ edited LSX Clone.22 female  ------------------------------------------------------AGGTGTGGCGGCTGTGTTTTGTATTGTAATTGATGCTCTGCGTAACCTCGGTTT

NCBI Gallus gallus (5.0)        CATCATTCAGCAAATGCTGCTGTCAATTATTGTAATGAGGCAGAGGATGTTGCGAGGGAGTGAAACATGAGGGCTGTGCTGCAGAACGGCTGCGGTTGGGCAGCTGTG
LSX WT male                     CATCATTCAGCAAATGCTGCTGTCAATTATTGTAATGAGGCAGAGGATGTTGCGAGGGAGTGAAACATGAGGGCTGTGCTGCAGAACGGCTGCGGTTGGGCAGCTGTG
FRZ edited LSX Clone.22 female  CATCATTCAGCAAATGCTGCTGTCAATTATTGTAATGAGGCAGAGGATGTTGCGAGGGAGTGAAACATGAGGGCTGTGCTGCAGAACGGCTGCGGTTGGGCAGCTGTG
```

Supplementary Fig. 13: Analysis of potential off-target mutations of *KRT75* gRNA.

a, List of five potential off-target sites with the highest CFD scores. The potential protospacer adjacent motifs (PAM) are in italics and mismatches between the gRNA and off-target sites are highlighted in bold. **b**, Genetic loci specification of these potential gRNA off-target sites. **c**, Alignments of the potential off-target loci from the NCBI *Gallus gallus* (5.0) genome, LSX PGCs, and the FRZ-edited LSX PGC clone. The sequencing primers are in blue and gDNA complementary sequence is in red (with the mismatches from the PMEL17 gRNA sequence on bold and underlined). Any changes from the NCBI reference genome sequence are in green.

Supplementary Methods

iCaspase9 vector design and construction

A 1.5 kb left targeting arm was PCR amplified using genomic DNA from Hy-line PGCs up to the *DAZL* stop codon. The middle fragment containing a 2A-GFP fragment was PCR amplified from an existing expression plasmid (DDX4-GFP). The right 1.5 kb targeting arm was PCR amplified from genomic DNA downstream from (and including) the *DAZL* stop codon. Gibson cloning was used to construct an initial *DAZL* left arm-2A\2A-GFP*DAZL* right arm targeting vectors. Equimolar quantities of DNA fragments were incubated with the Gibson HiFi DNA Assembly Master Mix enzyme (NEB) for 2 hours at 50 °C and transformed into bacteria for plasmid isolation and sequencing.

The human and chicken 2A-iCaspase9 sequences (Supplementary Note 1) were commercially synthesised (Invitrogen) with BamH1 restriction sites using plasmid pMSCV-F-del Casp9.IRES.GFP (Addgene) as a template and subcloned into the DAZL-GFP construct.

Guide design and CRISPR/Cas9 vector construction

CRISPR gDNAs were designed using CRISPOR gDNA design web tool (http://crispor.tefor.net) and CHOPCHOP gDNA design web tool. gDNA oligonucleotides were synthesised by Integrated DNA Technologies (IDT) and cloned into HF-PX459 V2.0 as previously described in [44]. The HF-PX459 V2.0 vector was used to express SpCas9-HF1 and sgRNA. Construction of the HF-PX459 V2.0 vector is previously described in [10]. All ssODN donors used were Ultramer DNA Oligonucleotides synthesised by IDT. ssODN and gRNA sequences are listed in the Supplementary Table 4 below. Microhomology-mediated end joining at the *PMEL17* guide double strand break site were predicted using inDelphi and RGEN Tools microhomology predictors [29,30].

Detection of genome editing events

To detect editing events genomic PCR was performed with corresponding specific primer sets (Supplementary Table 5). For *KRT75* screening of PGC clones, cells were lysed in QuickExtract DNA extraction solution (Epicentre) following manufactures instructions. 2 µl of this lysate or 100 ng of gDNA were used as PCR template for *KRT75* PCR amplification with Phusion High-Fidelity DNA polymerase (NEB) according to manufacturer's instructions. For *PMEL17* screening, an initial PCR was performed using 200 ng of genomic DNA as PCR template for amplification with FastStart Taq DNA Polymerase (Sigma-Aldrich) according to manufacturer's instructions. Subsequently, to identify an indel in *PMEL17* indel another PCR was performed, 100 ng of genomic DNA was used a template for amplification with PrimerSTAR GXL DNA polymerase (Takara Bio) according to manufacturer's instructions. All PCR products were directly sequenced by Sanger sequencing to detect edited genotypes. Digestion of the PCR products with Pfi1 detected the *PMEL17*$^{del+WAP}$ allele.

Selection and analysis of potential off-target sites

To detect any off-target gene editing events the NCBI chicken genome (gal/Gal5) was screening using the CRISPOR web tool to select the most probable off-target sites. Five candidates with the highest CFD scores were selected for analysis. For screening, a PCR was performed using 100 ng of genomic DNA as PCR template from PGC clones or blood samples from the indicated chickens and loci specific primers selected using NCBI Primer-BLAST web tool (see sequences below). The PCR amplification was performed with Phusion High-Fidelity DNA polymerase (NEB) according to manufacturer's instructions and PCR products were directly sequenced by Sanger sequencing and aligned to the respective NCBI sequence.

PCA plot

Genomic DNA was prepared from blood samples from G_1 chicks using cell lysis solution (Qiagen) containing RNAse A Solution (Sigma-Aldrich). Protein Precipitation Solution (Qiagen) was added and DNA was precipitated and resuspended. DNA from G_1 offspring and control (Hy-line Brown, iCaspase9, LSX, and WL Line 6) chicks were genotyped using a custom Cobb 60K Infinium Illumina array.

Immunofluorescence and Microscopy

Dissected gonad images were imaged on a Leica MZ8 light microscope. Gonad sections were visualized using a Zeiss LSM 710 inverted confocal microscope using standard 488 or 543 excitation. All mages were captured using Zen Black software (Zeiss). 15µm cryosections of gonads were washed with PBT for 30 min, and incubated with primary antibodies overnight. Sections were washed for one hour then incubated with secondary antibodies conjugated with Alexa- Fluor 546 (goat 1:500, Thermo Fisher Scientific) for one hour at room temperature. Samples were washed PBT for 30 min, and stained with Hoechst (Sigma) to visualize nuclei. Primary antibodies were anti-DDX4 antibody (rabbit 1:250, kindly gifted by Craig Smith (Monash University, Melbourne) or anti-active Casapase-3 (rabbit 1:250, BD Biosciences).

Western blot analysis of expression of HA and GFP in CIA gonadal PGC

Gonads from iCaspase9 or aviCaspase9 embryos at 10 days of incubation were examined for expression of GFP protein under fluorescent microscopy. The GFP^+ gonads and other tissues were dissected and GFP-negative gonads were used as controls. The gonads were lysed into RIPA buffer containing proteinase inhibitor. Protein (~75 mg) was resolved on 5-14% SDS-PAGE gel (Bio-rad) and trans-blotted onto PVDF membrane. Anti-HA tag (rabbit, 1:2000, Cell Signalling Tech.) and anti-GFP (mouse, 1:2000, Millipore) antibodies were used to probe the target proteins, anti-alpha tubulin (mouse 1:8000, Sigma-Aldrich) antibody was used as loading control. Secondary antibodies were anti-rabbit IgG, HRP-linked (goat 1:4000, Cell Signalling Tech.) and anti-mouse IgG, HRP-linked (goat 1:4000, Santa Cruz Biotech). To enrich for germ cells, gonads were also dissociated in parallel with collagenase/dispase enzyme (Sigma-Aldrich) and the cells were cultured in FAOT medium for 24 hours. The cells in

suspension were transferred to a new well on 24-well plate and further cultured for and additional 4 days. Suspension cells were harvested into RIPA buffer and used for western blots.

Flowcytometric analysis

Gonads were dissected from day 10 male or female embryos. A single cell suspension was prepared by dissociation of tissues in dispase/collagenase (Roche) for 10 min. The cells were immunostained using the dead cell marker dead cell marker Zombie Violet (Biolegend), SSEA-1 primary antibody (mouse 1:500, R&D system), followed by anti-mouse IgM conjugated with Alexa fluor-546 antibody (goat, 1:500, Thermofisher Scientific). The stained cells were analysed on BD LSR Fortessa analyser using Flowjo V10 software.

Statistical Analysis

All data was tested for statistical significance in Microsoft Excel 2016. P values were calculated using the one way ANOVA test and were considered significant at $p<0.05$ and highly significant at $p<0.001$.

Supplementary Table 4. Oligonucleotides

Used for editing	Description	Sequence	Length
Male Line 6 WL PGC line	DOW male repair template	GCATCCCCAGCCGCCAGTGGCACCACGCTCACCGTGGGGCTGCTCCTCATCGCCGCTGCTCTGGGCACCGCTGCCTACACCTACCGGTGAGCGGG	95 bp
Female Line 6 WL PGC line	DOW female repair template	GCATCCCCAGCCGCCAGTGGCACCACGCTCACCGTGGGGCTCCTCCTCATCGCCGCTGCTCTGGGCACCGCTGCCTACACCTACCGGTGAGCGGG	95 bp
Female LSX PGC line	FRZ template	TCCCCAGCTCCCTCATCCTTTCTCCATAGGAGAAGGTTTTCCTGGAAGAGTCAGCCCGGCTGCGGTTGGCAATGTCCTCATACTGCGCCTTCACCTCGGC	100 bp
Line 6 WL PGCs	*PMEL17* guide	GCTCACCGTGGGGCTGCTCT	20 bp
Hy-line PGCs	*DAZL* guide	GGCTTACTAAACTGAACTGT	20 bp
LSX PGCs	*KRT75* guide	GGCTTCAGCCCGGCTGCGGT	20 bp

PMEL17 targeting in female WL Line 6 PGCs the HDR donor template (DOW female repair template) was redesigned to include a 1 bp synonymous change (highlighted in red). The change was introduced within 2 bp of the Cas9 cut site and also introduces a novel restriction site, Eco24I (BanII), see underlined in table above.

Supplementary Table 5. PCR primers

Target	Primer name	Sequence	Product length
PMEL17	Pmel_exon10fwd	TGGCTGTGGCCAGCACCCACG	382 bp
	MM-532	GCAAACGCAGGGTAGCAC	
KRT75	KRT75_F1	AAGGCAGATGCATTGACCGA	657 bp
	KRT75_R1	CATCAACTGCCCAGGGACTC	
iCaspase9 screening	mm786	TGCCTGGTTGCTTTAATTTCCTC	1017 bp
	mm787	TGGAACAGGTAAAACAGAACACA	
aviCaspase9 screening	mm785	GTCGACGGTGTCTCTGTGAA	1813 bp
	mm787	TGGAACAGGTAAAACAGAACACA	
Recessive white locus	Diag05-nor-up	CAAAACCATAAATAGCACTGGAAATAG	481 bp
	Diag05-dw	TTGAGATACTGGAGGTCTTTAGAAATG	
	Diag05-cc-up	CCTCTGGCTCTATTTGACTACACAGT	345 bp
	Diag05-dw	TTGAGATACTGGAGGTCTTTAGAAATG	
GAPDH	c-gapdhF	CAGATCAGTTTCTATCAGC	700 bp
	c-gapdhR	TGTGACTTCAATGGTGACA	
GFP	GFP-F	ACGTAAACGGCCACAAGTTC	187 bp
	GFP-R	AAGTCGTGCTGCTTCATGTG	
KRT5	KRT5_F1	CCCACTAACCTTGTCCCCAA	443 bp
	KRT5_R1	TCTCTTGTGCAGTGCTGAGAT	
LHX2-NEK6	LHX2-NEK6_F1	TTCAGGGACTTGGAGCCTTC	447 bp
	LHX2-NEK6_R1	TGCACTCTCTGCCCGATTAG	
CUX	CUX1_F1	CAGGGAGGGTTTTCCGTACC	559 bp
	CUX1_R1	TGCTGTGCAGGCTGAGTTTA	
PPP1R3A-GPR85	PPP1R3A-GPR85_F1	AGAACACCACGCATTAGCCA	403 bp
	PPP1R3A-GPR85_R1	GACGCCAGATAGTCAGAGCC	
TCF7	TCF7_F1	GTCGTTCCTGCACTGCGATA	350 bp
	TCF7_F1	AACTTGTGGAAACCACGGCT	
ENSGALT00000075913.1/ ENSGALT00000055057.1	intronENSGAL_F1	TGCCAAGACTCCAGCACTAC	583 bp
	intronENSGAL_R1	CTTCTTGCCCCATGGCTGTA	
FGFR3- ENSGALT00000073982.1/ ENSGALT00000064614.1	FGFR3-ENSGAL_F1	CAACCGTCCACCTATCACCAA	393 bp
	FGFR3-ENSGAL_R1	GAGATGAGCCCAGCACAATG	
MTBP/MRPL13-MTBP	MRPL13-MTBP_F1	ATGCGTGTGGATGAGATACCC	362 bp
	MRPL13-MTBP_R1	TTGCCTGGGGTTGTTGTGAT	
PI4KA-UBE2L3	PI4KA-UBE2L3_F1	CTGCGTTCCTCACTGTCATC	418 bp
	PI4KA-UBE2L3_R1	GAGCTGGAGGGGTACCATAC	
SCARA5	SCARA5_F1	CTCTCTGAGTTGTGGCTGGG	365 bp
	SCARA5_R1	GCAGGGGACATCCATGAACA	

Supplementary Note 1. Sequences of iCaspase9 transgenes.

DNA sequence for human iCaspase9 transgene
(2A-iCaspase9-Bam H1 site mutated)
GGATCCGGAGCTACTAACTTCAGCCTGCTGAAGCAGGCTGGAGACGTGGAGGAGAACCCTGGACCT
ATGCTCGAGGGAGTGCAGGTGGAGACTATCTCCCCAGGAGACGGGCGCACCTTCCCCAAGCGCGG
CCAGACCTGCGTGGTGCACTACACCGGGATGCTTGAAGATGGAAAGAAAGTTGATTCCTCCCGGGA
CAGAAACAAGCCCTTTAAGTTTATGCTAGGCAAGCAGGAGGTGATCCGAGGCTGGGAAGAAGGGG
TTGCCCAGATGAGTGTGGGTCAGAGAGCCAAACTGACTATATCTCCAGATTATGCCTATGGTGCCAC
TGGGCACCCAGGCATCATCCCACCACATGCCACTCTCGTCTTCGATGTGGAGCTTCTAAAACTGGAA
TCTGGCGGTGGTTCCGGAGTCGACGGATTTGGTGATGTCGGTGCTCTTGAGAGTTTGAGGGGAAAT
GCAGATTTGGCTTACATCCTGAGCATGGAGCCCTGTGGCCACTGCCTCATTATCAACAATGTGAACTT
CTGCCGTGAGTCCGGGCTCCGCACCCGCACTGGCTCCAACATCGACTGTGAGAAGTTGCGGCGTCG
CTTCTCCTCGCTGCATTTCATGGTGGAGGTGAAGGGCGACCTGACTGCCAAGAAAATGGTGCTGGC
TTTGCTGGAGCTGGCGCGGCAGGACCACGGTGCTCTGGACTGCTGCGTGGTGGTCATTCTCTCTCAC
GGCTGTCAGGCCAGCCACCTGCAGTTCCCAGGGGCTGTCTACGGCACAGATGGATGCCCTGTGTCG
GTCGAGAAGATTGTGAACATCTTCAATGGGACCAGCTGCCCCAGCCTGGGAGGGAAGCCCAAGCTC
TTTTTCATCCAGGCCTGTGGTGGGGAGCAGAAAGACCATGGGTTTGAGGTGGCCTCCACTTCCCCTG
AAGACGAGTCCCCTGGCAGTAACCCCGAGCCAGATGCCACCCCGTTCCAGGAAGGTTTGAGGACCT
TCGACCAGCTGGACGCCATATCTAGTTTGCCCACACCCAGTGACATCTTTGTGTCCTACTCTACTTTCC
CAGGTTTTGTTTCCTGGAGGGACCCCAAGAGTGGCTCCTGGTACGTTGAGACCCTGGACGACATCTT
TGAGCAGTGGGCTCACTCTGAAGACCTGCAGTCCCTCCTGCTTAGGGTCGCTAATGCTGTTTCGGTG
AAAGGGATTTATAAACAGATGCCTGGTTGCTTTAATTTCCTCCGGAAAAAACTTTTCTTTAAAACATC
AGTCGACTATCCGTACGACGTACCAGACTACGCACTCGACCTCGACGGATCC

DNA sequence for chicken iCaspase9 transgene
(2A-aviCaspase9)
AGGGATCCGGAGCTACTAACTTCAGCCTGCTGAAGCAGGCTGGAGACGTGGAGGAGAACCCTGGA
CCTATGCTCGAGGGAGTGCAGGTGGAGACTATCTCCCCAGGAGACGGGCGCACCTTCCCCAAGCGC
GGCCAGACCTGCGTGGTGCACTACACCGGGATGCTTGAAGATGGAAAGAAAGTTGATTCCTCCCGG
GACAGAAACAAGCCCTTTAAGTTTATGCTTGGCAAGCAGGAGGTGATCCGAGGCTGGGAAGAAGG
GGTTGCCCAGATGAGTGTGGGTCAGAGAGCCAAACTGACTATATCTCCAGATTATGCCTATGGTGCC
ACTGGGCACCCAGGCATCATCCCACCACATGCCACTCTCGTCTTCGATGTGGAGCTTCTAAAACTGG
AATCTGGCGGTGGTTCCGGAGTCGACGGTGTCTCTGTGAATTGCAGACCAGCTAGGATGCATGCTA
GTGCATGCCAGGTGTACCAGCTGCGAGCAGACCCTTGTGGGCACTGCCTGATCTTCAACAATGTCAG
CTTCAGCAGAGACTCTGATCTGTCGACTCGAGCTGGCTCTGACATAGACTGTGAGAAGCTGGAGAA
GCGTTTCAGGTCCCTGTGCTTCCACGTCCGGACCCTGCGGAACCTCAAAGCTCAGGAAATTGATGTG
GAGCTGCGGAAGCTGGCGCGGCTCGACCACAGTGCCCTGGACTGCTGCCTCGTGGTCATCCTCTCCC
ATGGTTGCCAGACAAGCCATATTCAGTTTCCCGGAGGGATTTATGGAACAGATGGCAAAATCATTCC
AATCGAAAGGATTGTGAACTATTTCAATGGGTCCCAGTGCCCGAGTTTGAGAGGAAAACCCAAACT
CTTCTTCATCCAGGCCTGTGGAGGAGAACAAAAGGACCAAGGATTTGAGGTGGATTGTGAATCACC
CCAAGATGAAACTTGCCGACGTTCCATAGAGTCGGATGCGATTCCTTTCCAGGCTCCATCAGGGAAT
GAGGACGAGCCAGACGCCGTCGCCAGTTTGCCCACTCCTGGTGACATCTTGGTGTCCTATTCAACTT
TTCCAGGTTTTGTGTCCTGGAGGGACAAGGTGAGTGGCTCGTGGTACGTGGAAACCTTGGACAGCG
TACTGGAACATTACGCCCGTTCTGAAGACCTGCTTACCATGCTACTTCGGGTGTCAGACATCGTATCC
ACCAAGGGGAGGTACAAGCAGATCCCGGGCTGTTTCAACTTCCTTCGTAAAAAATTCTTCTTCCTGT
GCAAGGTCGACTATCCGTACGACGTACCAGACTACGCACTCGACGGATCCAA
